# Design and Algorithm Integration of High-Precision Adaptive Underwater Detection System Based on MEMS Vector Hydrophone

**DOI:** 10.3390/mi15040514

**Published:** 2024-04-12

**Authors:** Yan Liu, Boyuan Jing, Guojun Zhang, Jiayu Pei, Li Jia, Yanan Geng, Zhengyu Bai, Jie Zhang, Zimeng Guo, Jiangjiang Wang, Yuhao Huang, Lele Xu, Guochang Liu, Wendong Zhang

**Affiliations:** State Key Laboratory of Dynamic Measurement Technology, North University of China, Taiyuan 030051, China; b200612@st.nuc.edu.cn (Y.L.); yzflcm@163.com (B.J.); sz202106186@st.nuc.edu.cn (J.P.); jialinuc@163.com (L.J.); b20220613@st.nuc.edu.cn (Y.G.); s202306001@st.nuc.edu.cn (Z.B.); b20230611@st.nuc.edu.cn (J.Z.); s202206100@st.nuc.edu.cn (Z.G.); sz202206036@st.nuc.edu.cn (J.W.); sz202206068@st.nuc.edu.cn (Y.H.); sz202106168@st.nuc.edu.cn (L.X.); b200611@st.nuc.edu.cn (G.L.); wdzhang@nuc.edu.cn (W.Z.)

**Keywords:** MEMS vector hydrophone, DOA estimation, adaptive signal processing

## Abstract

Real-time DOA (direction of arrival) estimation of surface or underwater targets is of great significance to the research of marine environment and national security protection. When conducting real-time DOA estimation of underwater targets, it can be difficult to extract the prior characteristics of noise due to the complexity and variability of the marine environment. Therefore, the accuracy of target orientation in the absence of a known noise is significantly reduced, thereby presenting an additional challenge for the DOA estimation of the marine targets in real-time. Aiming at the problem of real-time DOA estimation of acoustic targets in complex environments, this paper applies the MEMS vector hydrophone with a small size and high sensitivity to sense the conditions of the ocean environment and change the structural parameters in the adaptive adjustments system itself to obtain the desired target signal, proposes a signal processing method when the prior characteristics of noise are unknown. Theoretical analysis and experimental verification show that the method can achieve accurate real-time DOA estimation of the target, achieve an error within 3.1° under the SNR (signal-to-noise ratio) of the X channel of −17 dB, and maintain a stable value when the SNR continues to decrease. The results show that this method has a very broad application prospect in the field of ocean monitoring.

## 1. Introduction

The positioning and identification of various types of undersea craft and underwater submarines in the ocean is an important task, and a key means for each country to design anti-submarine, escort, and other defense weapons. The real-time determination of underwater target orientation is one of the most important fields of marine safety monitoring and target detection. The precise orientation of underwater targets provides a research basis for hydrophone-based remote sensing detection by buoys. With few studies on the real-time determination of target orientation, a large number of studies have focused on post-processing of signals and adopting algorithms with extremely high computational complexity to improve the accuracy of target orientation estimation after acquisition. In DOA estimation, the high-resolution performance is mostly concerned, whereas these workflows are difficult to execute in hardware systems. Thus, many similar studies are difficult to be applied in practice [1,2]. In addition, there are studies on underwater high-resolution visual reconstruction, which can even convey high-definition images of a target, with the premise of needing to recognize the device and in close proximity to the target to be detected. When the distance is farther away from each other, it is necessary to rely on the detection device to determine the target’s orientation in advance, and then perform the reconstruction of the image after being close to the target [3,4]. In contrast to electromagnetic and light waves, sound is the only form of energy found so far in the oceans that can travel over long distances, so there is still a demand for hydroacoustic technology for long-range ocean target exploration. The underwater acoustic environment is characterized by strong noise and interference, rapidly variable signals in the time and frequency domains, and significant multipath propagation. Therefore, robust target orientation detection underwater is challenging [5,6]. The cilia MEMS bionic vector hydrophone has features of low cost, low power consumption, miniaturization, and good low-frequency performance, as well as higher sensitivity and wider bandwidth with the continuous research and improvement of the MEMS vector hydrophone, exhibiting excellent performance in the field of hydroacoustic detection [7,8]. In 2022, Zhu Shan et al. designed a noise measurement system based on a MEMS vector hydrophone for the measurement of radiated noise from underwater ships. The workflow of the system is presented: the raw data of signals were saved by the storage device during the acquisition process, and the data were read by the upper computer for the calculation of the ship’s radiated noise after acquisition. However, the main function of the system was signal storage, rather than the real-time processing of the hardware, especially the real-time judgment of target orientation [9]. The underwater detection system designed in this paper is capable of signal storage and real-time processing. On the one hand, the hardware system is internally integrated with a signal processing and orientation estimation algorithm to determine target orientation in real time, and on the other hand, stable and reliable raw data are stored for use in subsequent analysis.

An adaptive linear enhancer mentioned in Ref. [10] is based on the least mean squares (LMS) algorithm to adaptively update the filter tap coefficients, showing good real-time performance, which is favored in engineering applications [10]. This type of algorithm is widely employed in adaptive filtering, as it continuously adjusts the filter weights to minimize the mean squared error between the filter output and the desired output.

A variety of studies on the LMS algorithm have appeared after years of development, and have continuously improved the effect of adaptive linear enhancement [11,12]. The LMS algorithm has advantages of low computational complexity, good convergence in environments with smooth signals, and good stability when the algorithms are implemented using finite step size; therefor, the LMS algorithm provides the best stability and has the most widespread application among adaptive algorithms [13,14].

**Figure 1:**
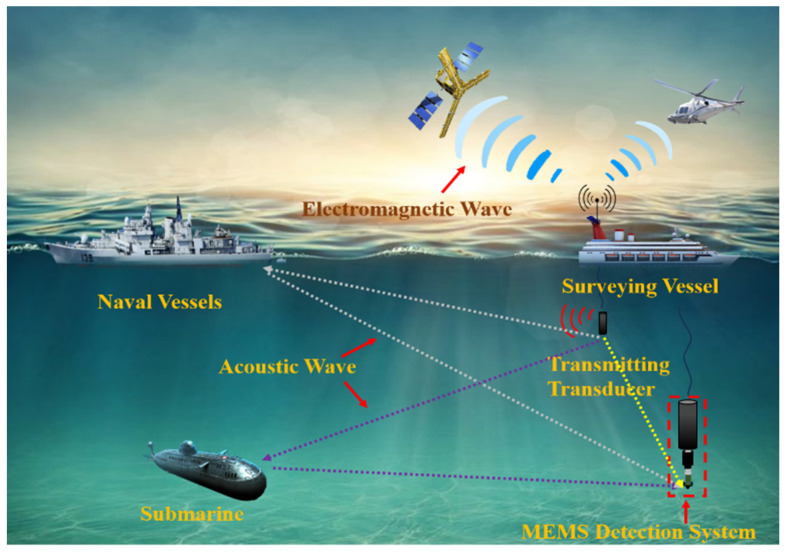
Diagram of underwater target orientation detection system in operation.

An adaptive filter applying the optimized LMS algorithm is proposed for the detection system designed in this paper, and the DOA estimation method of a single MEMS vector hydrophone is studied based on this adaptive filter. The method firstly performs a Fourier transform of a section of the input signal received by the MEMS vector hydrophone to obtain the frequency of the input signal, and then constructs a reference signal by taking the frequency corresponding to the peak of the spectrum in the input signal as the frequency of the reference signal. The input signal produces the output signal by the filter, which is compared to the reference signal to form the error signal. In this paper, the parameters of the filter are adjusted by the adaptive iterative method with the criterion of minimizing the mean-square error of the error signal, and the filtered target signal is finally output. This filtering method allows the filtering of signals when the statistical characteristics of the original signal are unclear. The method in this paper is particularly applicable in bistatic active detection, where a different reference signal can be automatically matched at the receiving terminal when the signal frequency is changed at the transmitting terminal of the transducer [15]. Figure 1 shows the workflow of the detection system. The red waveform represents sound waves, the blue waveform represents electromagnetic waves, the green dashed line represents the direct wave of sound waves, and the gray and purple dashed lines represent the reflected waves of sound waves.

The novelty and the main task of this paper are to propose the LMS adaptive signal processing method for the miniature MEMS vector hydrophone detection system designed in this paper and to achieve this function in a hardware system, which automatically generates a reference signal and updates the filtering parameters to filter this signal, provided that the statistical characteristics of the original signal are not clear. In this paper, the filtering effect under different SNRs and the DOA estimation error are simulated by analog signal MATLAB to determine appropriate filter parameters. The standing wave tube and reservoir experiments verify the hardware system angle real-time calculation and system raw data storage function. The experimental results show that the system designed in this paper and the adaptive algorithm integrated internally can realize the DOA estimation under different frequencies and different SNRs, and the orientation error is less than 3° under the premise of the unknown statistical characteristics of the original signal. The achievements in this paper are of great reference significance for the design of software and hardware systems for the underwater target orientation estimation of underwater target detection equipment, such as sonobuoys, UUVs, and AUVs.

## 2. Principles and Methods

### 2.1. Design of MEMS Detection System

**Figure 2:**
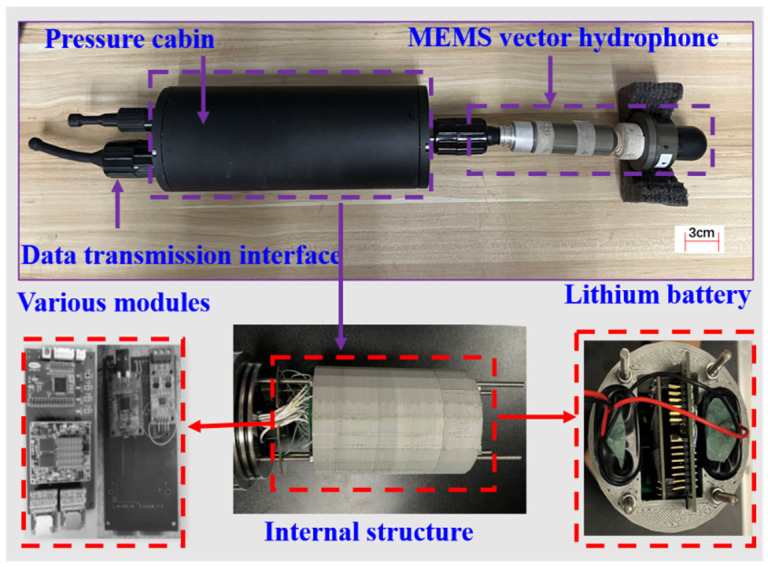
General structure of the detection system.

The MEMS vector hydrophone target orientation detection system can save the information detected by vector hydrophones and attitude sensors, and integrates adaptive filters and orientation estimation algorithms in the detection system to be capable of real-time target orientation estimation. Figure 2 shows the general structure of the detection system, comprising a pressure-resistant compartment, a data transmission interface, and a MEMS vector hydrophone, wherein the pressure-resistant compartment contains a circuit board, a lithium battery, and a fixing device. The purple dashed line represents the physical diagram of the testing system, the red dashed line represents the internal structure of the system, and the content indicated by the arrows represents the names of the system structures or enlarged portions of the diagram. The system adopts the low power consumption design, and the lithium battery in the pressure-resistant chamber can ensure that the system can operate continuously underwater for more than 20 h. In order to accurately record underwater sound, the sampling frequency is set to 10 samples/s, and the whole channel is equipped with a high-precision clock to realize synchronous sampling.

**Figure 3:**
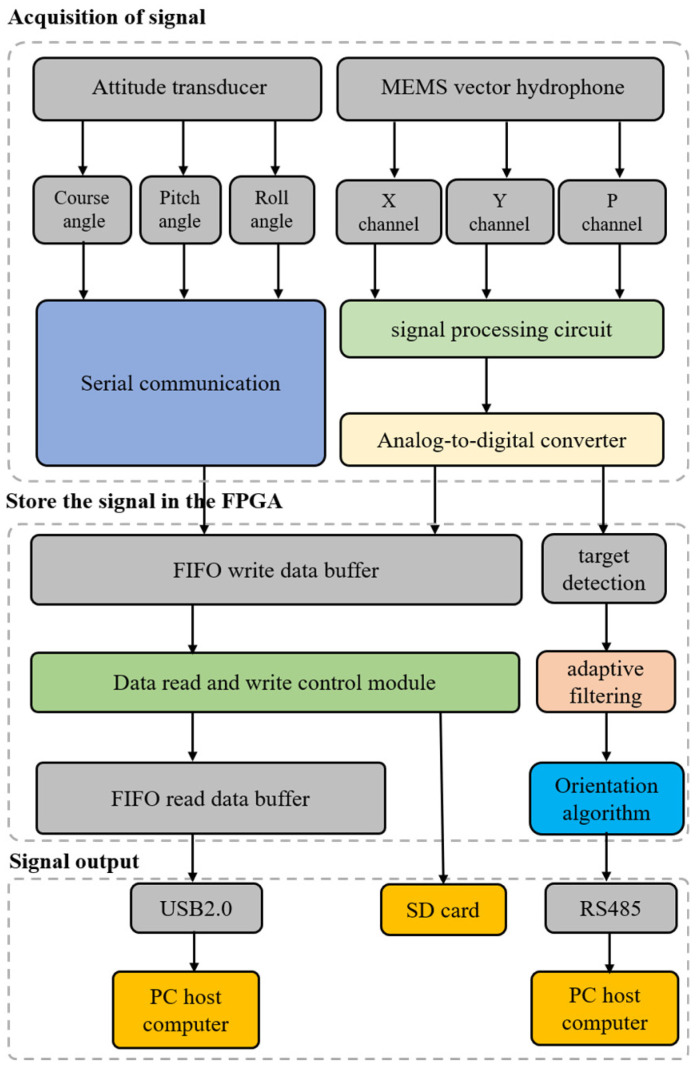
Workflow of the detection system.

Figure 3 provides the complete workflow of the detection system. The output of the three signals from the vector hydrophone are amplified and filtered by the front signal conditioning circuit and then digitally converted by the analog to digital (AD) module. At the same time, the attitude sensor transmits the attitude information of roll angle, pitch angle, and heading angle via the serial port. The data from the two sensors enter the field-programmable gate array (FPGA) and are written to the SD card for storage via the first in, first out (FIFO) cache, and finally read to the upper computer via the USB interface. At the same time, FPGA carries out the online data processing, detects and filters the signals of the received MEMS vector hydrophone data, calculates the relative angle of the target using the orientation estimation algorithm, and finally sends the calculation results via RS485 to complete the real-time estimation of the hardware target orientation.

### 2.2. Sensing Principle of MEMS Vector-Sensitive Probe

The MEMS vector hydrophone selected for this system is a sensor that mimics the movable cilia inside the neural mound of fish to sense the acoustic signals in the water, which is inspired by the lateral line organs of fish. The principle of acoustic signal perception in fish is as follows: acoustic waves make the cilia swing via the mucus, and then the sensory cells convert the acoustic signals into biological ones, which are then transmitted to the medulla oblongata of the fish via the efferent nerves, and the external acoustic signals can be ultimately perceived [16]. With the features of small size, high sensitivity, and good low-frequency response, the MEMS bionic vector hydrophone adopts a bionic column to simulate the sensory cilia of the lateral line organ and piezoresistor to simulate the sensory cell.

**Figure 4:**
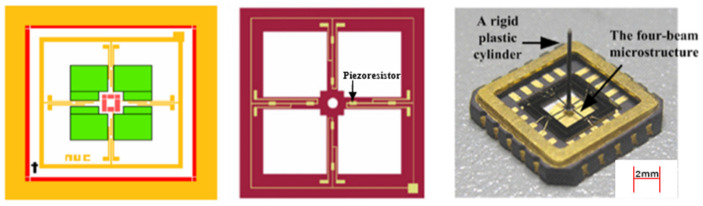
The cross-beam microstructure of the sensor and physical drawing.

The sensor mainly consists of a chip and a transmissive cap. The transmissive mate-rial is butyl rubber or polyurethane, filled with silicone oil. Eight equal-strain piezoresistors are distributed on the four-beam structure by diffusion technique and connected to two Wheatstone bridges. The distribution and connection of the piezoresistors are shown in Figure 4. When there is no acoustic signal, the Wheatstone bridges are in equilibrium; when there is an acoustic wave, the cilia swing leads to the deformation of the silicon cantilever beam structure, thereby causing a change in the resistance value of the piezo-resistor, destroying the equilibrium of the Wheatstone bridges, generating the corresponding voltage signal, and completing the conversion from acoustic signals to electrical signals via the two Wheatstone bridges to achieve the detection of hydroacoustic signals [2]. 

**Figure 5:**
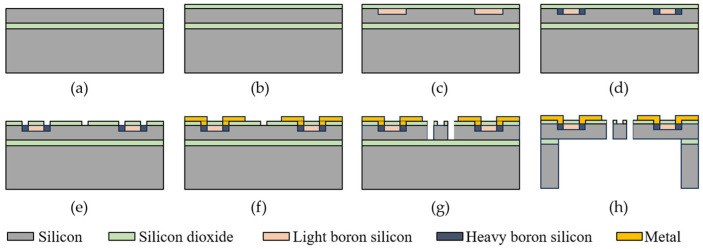
MEMS vector hydrophone fabrication process.

Using standard MEMS processes to fabricate the hydrophone chip, Figure 5 shows the complete process flow [17]:

(a) The wafer is subjected to acid and alkaline cleaning to remove surface organic matter, particulate contaminants, and metal impurities. Subsequently, it is rinsed multiple times with deionized water and dried with nitrogen to meet the cleanliness requirements for thermal oxidation and deposition processes on the wafer.
(b) Plasma-enhanced chemical vapor deposition (PECVD) is used to form a 20 nm thick oxide layer on the wafer surface, aiming to reduce lattice damage and achieve electrical isolation of the metal.
(c) The first photolithography is performed on the front side to inject B ions into the pressure resistance region, with an injection dose of 4.0 × 10^14^ cm^2^ and an injection energy of 80 keV. Subsequently, high-temperature annealing is conducted in a nitrogen environment at 1050 °C for 120 min.
(d) The second photolithography is carried out on the front side to inject B ions into the heavily doped region, with an injection dose of 3 × 10^15^ cm^2^ and an injection energy of 110 keV. Then, annealing is performed at 1000 °C for 30 min.
(e) The third photolithography on the front side utilizes reactive ion etching (RIE) to etch the oxide layer, exposing the heavily doped silicon region and the center positioning hole.
(f) The fourth photolithography on the front side involves depositing aluminum metal on the surface using sputtering technology. Subsequently, metal patterning is achieved using a lift-off process. Then, alloy annealing is conducted at 200 °C to form ohmic contact between the metal and the heavily doped region. At this point, the pressure resistance structure is formed.
(g) The fifth photolithography on the front side employs RIE technology to etch the deposited silicon oxide and device layer silicon, forming the four-beam-central block area.
(h) The sixth photolithography on the back side employs deep reactive ion etching (DRIE) technology to etch the back chamber. Then, the buried oxide layer is etched using HF buffer solution (BOE) to release the four-beam-central block structure.

**Figure 6:**
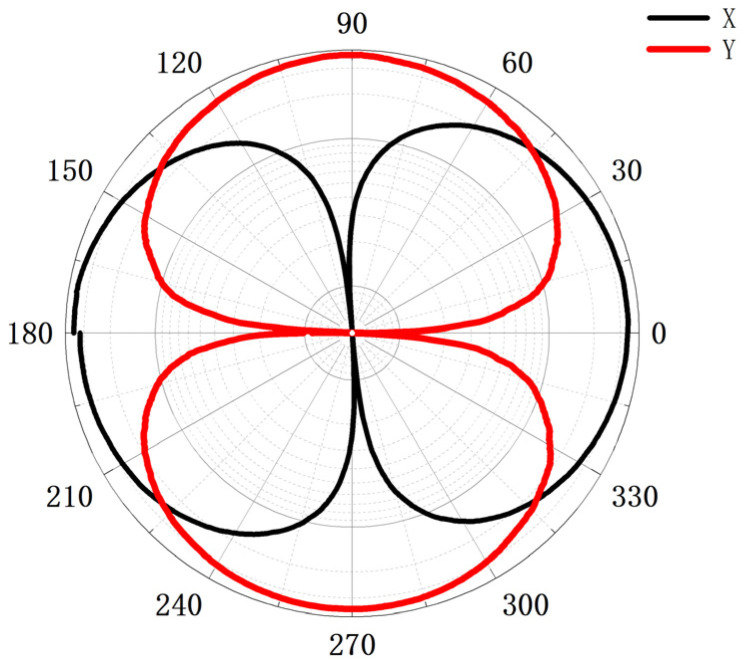
Diagram for directivity of the vector hydrophone.

The sensitivity of the vector channel of the hydrophone in this system is −175.4 dB@1 kHz (0 dB~1 V/µPa), and the scalar sensitivity is −181 dB (20–1000 Hz) (0 dB~1 V/µPa). The hydrophone used in this article remains consistent with the current leading level. Table 1 is a comparison of hydrophone performance. Furthermore, the sensor boasts a gain magnitude of 54 dB [2,18]. Figure 6 shows the test results of the sensor’s directivity at 315 Hz, and the calibration results show that the sensor has the directivity of the two vector channels in the shape of “8”, and the depth of concavity is more than 40 dB, with good directivity and consistency. 

The sensitivity of the MEMS sensors constrains the performance of the system, as it directly influences their capability to detect changes in environmental parameters. Lower sensitivity may result in a decrease in SNR, rendering them ineffective in capturing and distinguishing subtle signal variations, thereby impacting the accuracy and stability of the sensor. Moreover, inadequate sensitivity may limit the applicability of the testing system in certain scenarios, particularly those requiring high-precision measurements. The sensitivity of MEMS sensors also affects the system’s dynamic range. If sensitivity is low, it may hinder the sensor from accurately detecting or measuring signal changes within a wide range, thereby restricting the system’s dynamic performance. Hence, when designing testing systems, it is crucial to consider the limitations in sensitivity to ensure that the system meets the performance requirements of specific application scenarios.

### 2.3. LMS Adaptive Signal Processing Method and Improvements

The LMS algorithm is the most widely used adaptive filtering algorithm in practice, which can be attributed to its simplicity and robustness to signal statistics [19,20].

**Figure 7:**
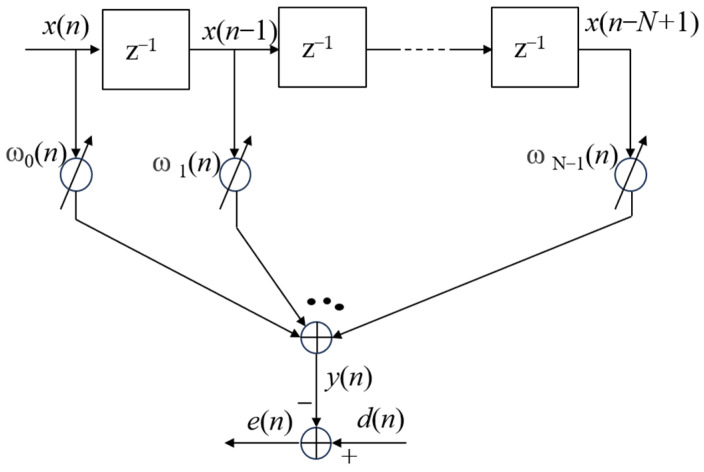
Workflow of the LMS algorithm.

Figure 7 gives an N-tap transversal adaptive filter. Solid line arrows indicate one-way associations, arrows containing dashed lines and ellipses indicate omissions, and Z^−1^ represents the next input. When there is a single frequency signal input, assume that the input of the filter is ***x***(*n*), the weight coefficient is ***w***(*n*), define ***x***(*n*) and ***w***(*n*) as column vectors, i.e.,

(1) $$x(n)={\left[x(n)\;x(n-1)\;\cdots \;x(n-N-1)\right]}^{T}$$

(2) $$w(n)={\left[w_{0}(n)\;w_{1}(n)\;\cdots w_{N-1}(n)\right]}^{T}$$

where superscript T stands for transpose. Suppose the desired output of the filter is *d*(*n*), the difference (error) is *e*(*n*), and the filter output *y*(*n*) is

(3) $$y(n)=\sum_{i=0}^{N-1}w_{i}(n)x(n-i)$$

They are all assumed to be real-valued sequences. The tap weights *w*_0_(*n*), *w*_1_(*n*), …, *w_N_*_−1_(*n*) are selected, so that the difference (error)

(4) e(n)=d(n) −y(n)

is minimized in some sense. The traditional LMS algorithm is a random implementation of the steepest descent algorithm, and the iteration of the weight vector can be expressed as

(5) $$w(n+1)=w(n)-\mu \nabla e^{2}(n)$$

where *μ* is the algorithm step-size parameter and ∇ is the gradient operator defined as the column vector.

(6) $$\nabla ={\left[\frac{\partial }{\partial \omega_{0}}\;\frac{\partial }{\partial \omega_{1}}\;\cdots \;\frac{\partial }{\partial \omega_{N-1}}\right]}^{T}$$

It is noted that the *i*th element of the gradient vector $\nabla e^{2}(n)$ is

(7) $$\frac{\partial e^{2}(n)}{\partial \omega_{i}}=2e(n)\frac{\partial e(n)}{\partial \omega_{i}}$$

Substituting Equation (4) into the last factor on the right side of Equation (7), and it is noted that *d*(*n*) is independent of *w_i_*, we obtain

(8) $$\frac{\partial e^{2}(n)}{\partial \omega_{i}}=-2e(n)\frac{\partial y(n)}{\partial \omega_{i}}$$

Substituting *y*(*n*) in Equation (3), we obtain

(9) $$\frac{\partial e^{2}(n)}{\partial \omega_{i}}=-2e(n)x(n-i)$$

Using Equations (6) and (9), we obtain

(10) $$\nabla e^{2}(n)=-2e(n)x(n)$$

Substituting this result in Equation (5), we obtain

(11) w(n+1)=w(n)+2μe(n)x(n)

This is known as LMS recursion. It is a simple procedure for recursively adapting the filter coefficients after the arrival of each new input sample ***x***(*n*) and its corresponding desired output sample *d*(*n*). Equations (3), (4) and (11) specify, in sequence, the three steps required to accomplish each iteration of the LMS algorithm. Equation (3) is known for filtering to obtain the filter output. Equation (4) is used to calculate the estimation error. Equation (11) is the tap-weight adaptive recursion [21,22].

The mean square value of the error signal is

(12) $$\xi \left(n\right)=E\left[d^{2}(n)\right]+w^{T}(n)E\left[x(n)x^{T}(n)\right]w(n)-2E\left[d(n)x^{T}(n)\right]w(n)$$

The adaptive linear combiner follows the criterion of minimizing the mean square value of the error signal.

The autocorrelation matrix *Rxx* = *E*[***x***(*n*) ***x***^T^(*n*)] of the input signal is

(13) $$Rxx=\left[\begin{array}{lll} E\left[x(n)x(n)\right] & E\left[x(n)x(n-1)\right] & \cdots E\left[x(n)x(n-L)\right]\\ E\left[x(n-1)x(n)\right] & E\left[x(n-1)x(n-1)\right] & \cdots E\left[x(n-1)x(n-L)\right]\\ E\left[x(n-L)x(n)\right] & E\left[x(n-L)x(n-1)\right] & \cdots E\left[x(n-L)x(n-L)\right] \end{array}\right]$$

The intercorrelation matrix *Rxd* = *E*[*d*(*n*) ***x***(*n*)] of the desired and input signals is

(14) $$Rxd=E\left[\;d\left(n\right)x\left(n\right)\;d\left(n\right)x\left(n-1\right)\;\cdots d\left(n\right)x\left(n-L\right)\right]\;$$

Then, the mean square error (MSE) is

(15) $$\xi \left(n\right)=E\left[d^{2}(n)\right]+w^{T}(n){R_{xx}}^{T}w(n)-2{R_{xd}}^{T}w(n)$$

This shows that the MSE is a quadratic function of the weight vector ***w***(*n*), which is a parabolic surface concave in the middle, and a function with a unique minimum [23]. The best weight vector can be obtained by differentiating the weight coefficient of Equation (14) through the fastest descent method, thus obtaining the minimum mean square error as shown in Equation (16), wherein ***w****_opt_* = *R_xx_*^−1^ *R_xd_*.

(16) $$\xi {\left(n\right)}_{min}=E\left[d^{2}(n)\right]-{R_{xd}}^{T}w_{opt}$$

With the noise reference signal unknown, to effectively handle target frequency signals, we employed a method called inverse extraction. Specifically, we first performed a Fast Fourier Transform (FFT) on the input signal. This step allowed us to convert the signal from the time domain to the frequency domain, enabling us to observe the distribution of the signal across different frequencies. Upon confirming the signal frequency through FFT, in the spectrum plot, we selected the frequency with the highest peak, identifying it as the frequency of the target signal, and retained the main frequency component for Inverse Fast Fourier Transform (IFFT), thereby generating a reference signal.

Next, we multiplied the constructed reference signal by a specific filter weighting coefficient (denoted as ***w***(*n*)), generating an output signal. The disparity between this output signal and the original input signal still represents the presence of noise, which is the error signal we aimed to eliminate. To minimize this error as much as possible, we need to engage in an iterative process, continuously adjusting the filter weighting coefficients to make the output signal closer to the target signal. This adjustment process is based on the principle of minimizing error, meaning we strive to make the disparity between the output signal and the target signal as small as possible.

**Figure 8:**
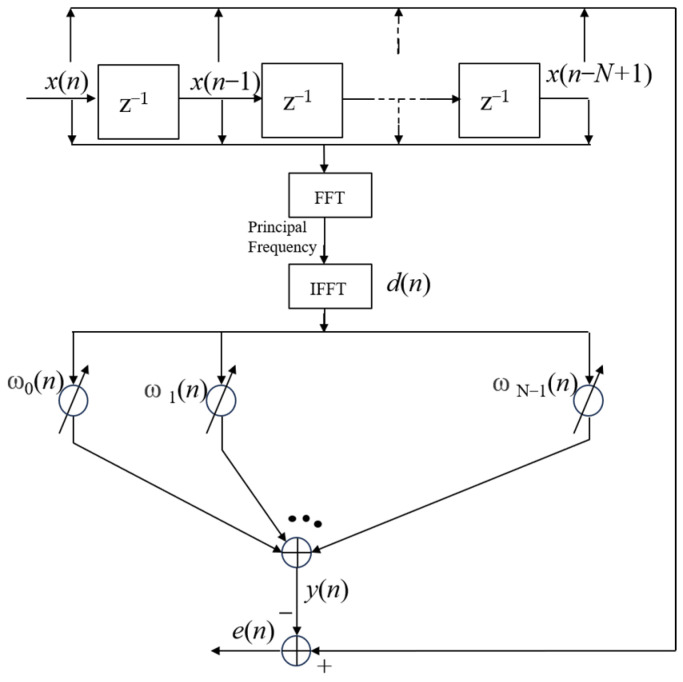
Optimized LMS algorithm workflow.

Through such steps, we ultimately succeeded in extracting the target signal, accomplishing the precise extraction and separation task of signals. This process is elaborately illustrated in Figure 8, enabling readers to gain a clearer understanding of the methodology we adopted and its specific implementation steps. The symbols in this figure have the same meanings as those in Figure 7.

This method is equivalent to considering the target signal at a fixed frequency as the input signal and the original input signal as the noise reference signal; the output signal at this point is the product of the weight coefficients and the desired signal after multiple iterations. This is advantageous since when the target signal is sinusoidal, especially in bistatic or multi-base active detection, a reference signal can be automatically generated that is valid for sensors with different frequency responses, without updating the reference signal each time. The adaptive filter has advantages such as a simple algorithm, easy implementation, low complexity of the algorithm, and the ability to suppress the side-lobe interference. However, there is also the drawback of slow convergence. The filter coefficients are updated in a point-by-point manner; thus, if the input signals are correlated, it would lead to the transmission of the gradient noise generated in the previous iteration to the next iteration, resulting in repeated transmission of the error, slower convergence, and poorer tracking performance. Moreover, another feature of the algorithm is equally important from the perspective of implementation, that is, its stable and robust performance under different signal conditions. In the following section, the simulation is carried out for input signals with different SNRs for different step sizes and different numbers of snapshots.

The step size *μ* is a physical quantity characterizing the iteration speed. It can be seen from the LMS algorithm that the larger the quantity is, the shorter the adaptive duration is, the faster the adaptive process is, but the greater the misalignment it causes. When it is larger than 1/*λ_max_*, the system disperses, whereas the smaller the value is, the more stable the system is, and the smaller the misalignment is, but the adaptive process is also correspondingly prolonged, $\lambda_{max}$ is the maximum eigenvalue of *R_xx_*. Therefore, the selection of the step size *μ* should be based on the requirements of the entire system, and the adaptive duration should be minimized while meeting the accuracy requirements. In order to test the filtering performance of the method proposed in this paper under the conditions of small number of snapshots and low SNR, and to provide some recommendations for the selection of parameters, the following three simulations were designed to analyze and visualize the features of the method in this paper. The SNR and the RMSE (root mean square error) of the filtered signal are used in the simulation as the criteria for the filtering effect: the higher the SNR and the smaller the RMSE of the output signal are, the higher is the quality of the recovered signal.

**Figure 9:**
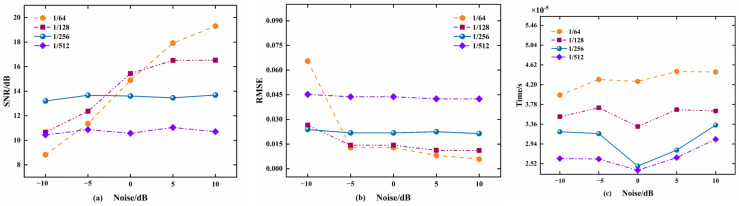
Simulation experiments with different time steps and SNR: (**a**) SNR of the output signal; (**b**) RMSE of output signal; (**c**) convergence time.

Simulation 1: Gaussian white noise with different power was superimposed on the original sinusoidal signal *x* = sin(2 × pi × 300 × t), the noise power was −10 dB, −5 dB, 0 dB, 5 dB, and 10 dB, and the number of snapshots was N = 3000, SNR, RMSE. The convergence times of the output signal with the time step *μ* of 1/64, 1/128, 1/256, and 1/512, respectively, were simulated by MATLAB, and the experimental results are shown in Figure 9. The results show that the SNR of the output signal is higher and can be stabilized, the RMSE is lower and stabilized, and the convergence time is faster when the time step *μ* = 1/256.

**Figure 10:**
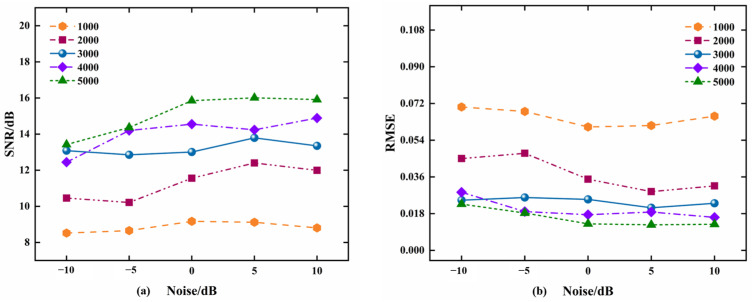
Simulation experiments with different snapshot numbers and SNR: (**a**) SNR of the output signal; (**b**) RMSE of the output signal.

**Figure 11:**
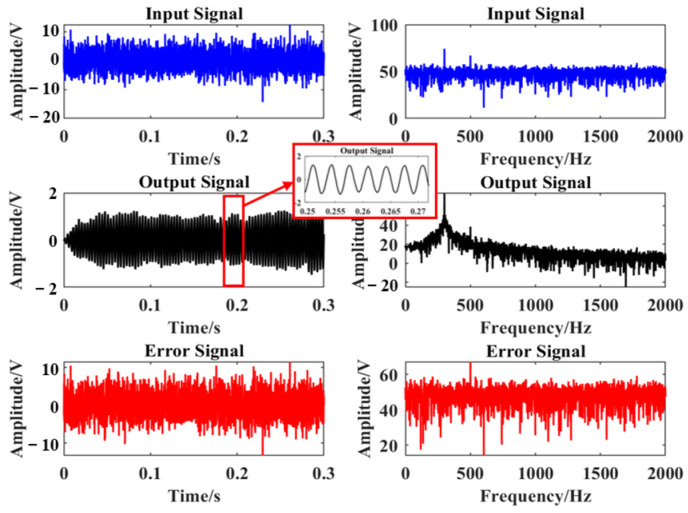
Signal processing results at noise of −10 dB.

Simulation 2: Gaussian white noise with different power was superimposed on the original sinusoidal signal *x* = sin(2 × pi × 300 × t), and the noise power was −10 dB, −5 dB, 0 dB, 5 dB, and 10 dB, respectively, and the time step *μ* = 1/256, SNR, and RMSE of the output signal when the number of snapshots was 1000, 2000, 3000, 4000, and 5000, respectively, were simulated by MATLAB R2018b. The experimental results are shown in Figure 10. The results show that the larger the number of snapshots, the larger the SNR and the smaller the RMSE of the output signal, when the number of snapshots, N, is greater than or equal to 3000, the output signal has a higher SNR and a lower RMSE, and both of them can keep stable with the increasing noise power of the input signal.Simulation 3: Gaussian white noise of −10 dB was superimposed on the original sinusoidal signal x = sin(2 × pi × 300 × t). According to the simulation results of Simulation 1 and 2 above, the number of snapshots N = 3000 and the time step *μ* = 1/256 were selected, and the output results of the input signals after this paper’s adaptive filter were simulated by MATLAB. Figure 11 shows the time-domain and spectrograms of the original input signal, output signal, and error signal, respectively. It can be clearly observed that the original signals have better recovery effect after this paper’s method at lower noise power. By virtue of the analysis in this section, it can be known that when the parameters are selected randomly, it may cause low SNR and large RMSE of the output signal, further reducing the signal recovery effect. Thus, the time step *μ* = 1/256 and the number of snapshots N = 3000 were selected in the subsequent data processing and hardware real-time processing procedures. 

### 2.4. DOA Estimation and Simulation

The average sound intensity method involves integrating the acoustic pressure after multiplying it with the vibration velocity, and then calculating the angle according to the formula. The average sound intensity flow *I_x_*, *I_y_*, respectively, is

(17) $${\bar{I}}_{x}=\bar{p(t)v_{x}(t)}=\bar{x^{2}(t)}\cos\theta +\bar{n_{p}(t)n_{x}(t)}+\;\bar{n_{p}(t)x(t)}\cos\theta +\bar{nx(t)x(t)}$$

(18) $${\bar{I}}_{y}=\bar{p(t)v_{y}(t)}=\bar{x^{2}(t)}\sin\theta +\bar{n_{p}(t)n_{y}(t)}+\;\bar{n_{p}(t)x(t)}\sin\theta +\bar{nx(t)x(t)}$$

*n_p_*(*t*), *n_x_*(*t*), *n_y_*(*t*) and *x*(*t*) can be neglected at high signal-to-noise ratios since they are independent of each other. Only the first term in Equations (17) and (18) is dominant and can be simplified as

(19) $${\bar{I}}_{x}=\bar{p(t)v_{x}(t)}=\bar{x^{2}(t)}\cos\theta +\bar{n_{p}(t)n_{x}(t)}+\Delta x$$

(20) $${\bar{I}}_{y}=\bar{p(t)v_{y}(t)}=\bar{x^{2}(t)}\sin\theta +\bar{n_{p}(t)n_{y}(t)}+\Delta y$$

where ∆*x* and ∆*y* are extremely small values that can be ignored. Hence, the horizontal azimuth angle *θ* of the acoustic source can be estimated using Equation (21).

(21) $$\hat{\theta }=\arctan\frac{\bar{p(t)v_{y}(t)}}{\bar{p(t)v_{x}(t)}}$$

Beam forming is a method of determining the spatial directivity by weighting and processing the signals of each channel using the “8”-shaped dipole directivity of the vector hydrophone. The three channels of acoustic field information collected by the hydrophone were weighted and summed, the vibration velocity signals in *x* and *y* directions were multiplied by the weighted amount of cosα and sinα, and the weighted amount of acoustic pressure signals was 1. It can focus the receiving direction of the hydrophone on a direction that is equivalent to the beam. Rotating the beam to find the maximum peak is target orientation [24,25,26].

The directivity of the combination of physical quantities can be characterized by taking the vibration velocity and acoustic pressure signals as an example. The acoustic pressure channel is omnidirectional, and the vibration velocity channel has an “8”-shaped directivity, which causes two angles of the target with a 180° difference in the estimation of the azimuth angle, and the true orientation of the target cannot be determined. By different weighted combinations of the acoustic pressure and the vibration velocity, the form of a unipolar directivity can be created. Two mutually orthogonal vibrational velocity components were weighted to generate a new form of vibrational velocity combination (Equation (22)). Take the following four representative combinations as an example: *v_c_*(*t*), *v_c_*(*t*) × *v_c_*(*t*), (*p*(*t*) + *v_c_*(*t*))^2^, *p*(*t*) + *v_c_*(*t*) × *v_c_*(*t*), the directivity is expressed as in Equations (22)–(25):

(22) $$v_{c}(t)=v_{x}(t)\cos\varphi +v_{y}(t)\sin\varphi =v(t)\cos(\theta -\varphi )$$

(23) $$v_{c}(t)\cdot v_{c}(t)=v^{2}(t)\cos^{2}(\theta -\varphi )$$

(24) $${\left(p(t)+v_{c}(t)\right)}^{2}={\left(1+\cos(\theta -\varphi )\right)}^{2}x^{2}(t)$$

(25) $$\left(p(t)+v_{c}(t))\cdot v_{c}(t)=(1+\cos(\theta -\varphi ))\cdot \cos(\theta -\varphi )\cdot x^{2}(t\right)$$

In the above equations, *v_c_*(*t*) shows “8”-shaped directivity, *v_c_*(*t*) × *v_c_*(*t*) represents narrow splayed directivity, (*p*(*t*) + *v_c_*(*t*))^2^ represents heart-shaped directivity, and *p*(*t*) + *v_c_*(*t*) × *v_c_*(*t*) represents tadpole-shaped directivity. The first two combinations have bi-lateral directionality, which can cause blurring in the port and starboard, while the latter two combinations present unipolar characteristics to suppress the anisotropic noise interference that is opposite to the target direction. In this paper, the combination of (*p*(*t*) + *v_c_*(*t*)) × *v_c_*(*t*) was adopted as the beam forming algorithm [27,28,29].

**Figure 12:**
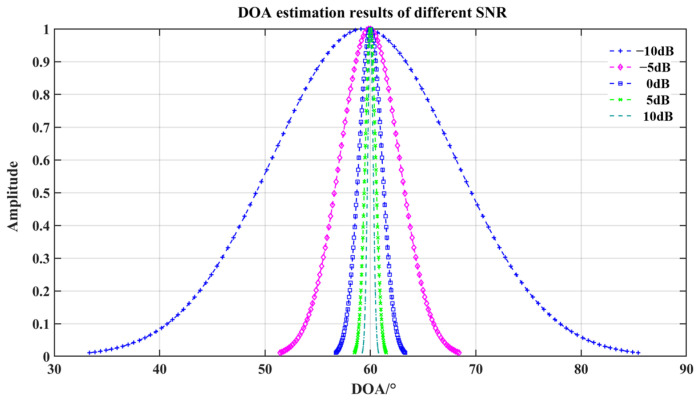
DOA estimation results at different SNRs.

In this paper, the DOA estimation results at different SNRs were simulated. The input signal frequency was 300 Hz, the original input signal was *x* = sin(2 × pi × 300 × t), the input angle was 60°, the number of samples was 3000, and the DOA estimation simulation was performed for 100 times after adding the noises with SNRs of 10 dB, 5 dB, 0 dB, −5 dB, −10 dB. The results of 5000 Monte Carlo simulations are shown in Figure 12. The DOA estimation results show Gaussian distribution. The estimated mean, standard deviation, and their respective confidence intervals (CI) are shown in Table 2 where the error in the mean value of the DOA estimation is less than 1°, and that in the CI is less than 3°. The simulation results show that the method is still effective for DOA estimation at a low SNR.

## 3. Experiment

### 3.1. Indoor Experiment

**Figure 13:**
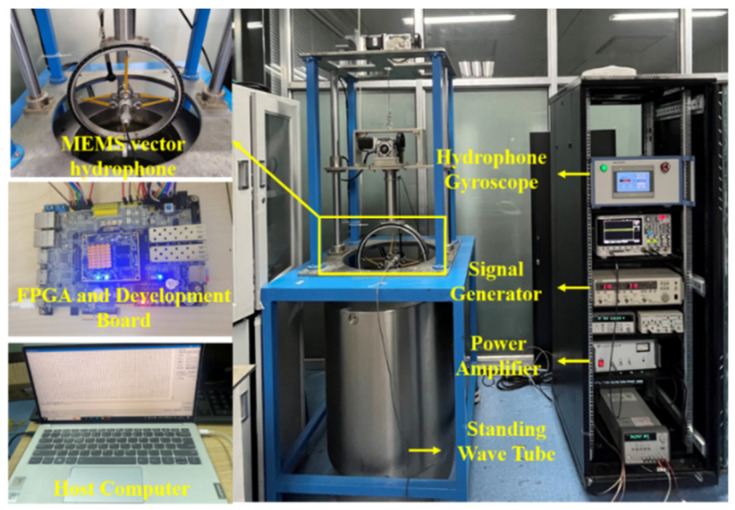
Sensor calibration and indoor test equipment.

After the hardware circuits for the main aspects of acquisition and storage, optimized LMS adaptive filtering, and real-time computation of target orientation angle are designed, the system functionality is firstly verified in the laboratory. Figure 13 shows the sensor calibration and indoor test equipment. The experiments for sensitivity calibration and angle real-time output of the MEMS vector hydrophone are performed in the standing wave tube. The main equipment used in the experiments include: standing wave tube, signal generator, power amplifier, gyroscope, and test system. The standard hydrophone is used as a reference for sensor calibration, the sensitivity of the hydrophone used in this experiment is −175 dB@1 kHz measured by the comparative calibration method, and the vector channel had good directivity, which meets the conditions of the angle real-time test.

**Figure 14:**
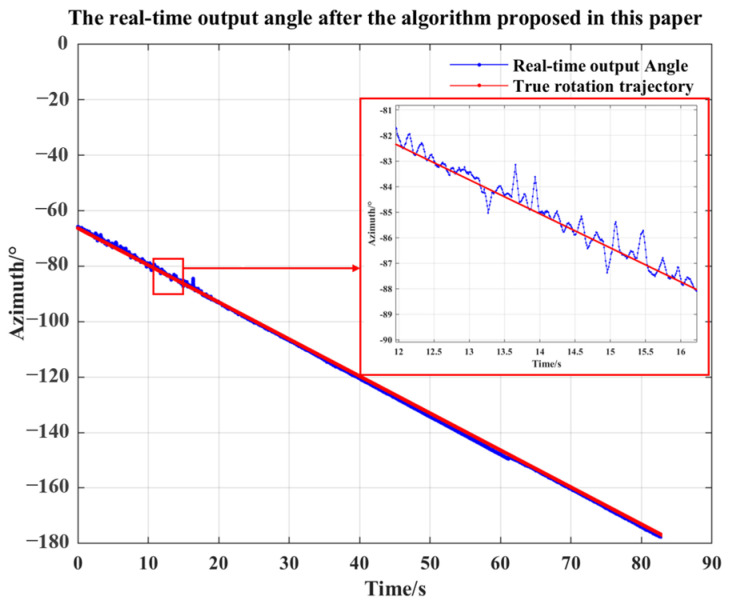
Angle real-time output results.

The test procedures are as follows: the hydrophone is suspended on the gyroscope, the sensor is lowered to the standing wave tube 46 cm from the acoustic source by the control system, a sinusoidal signal with a signal frequency of 210 Hz is emitted via the signal generator, and Gaussian white noise with an SNR of −10 dB is added to simulate the environment with large noise. At the same time, the gyroscope is rotated by a certain angle to change the angle of the acoustic source relative to the sensor, the angular value of the acoustic source detected by the sensor is output by the test system, and the rotational speed of the gyroscope is set to 80°/min. The sensor receives the acoustic signal at a rate of 10 k/s, the number of snapshots for the LMS filter as well as the average sound intensity algorithm to calculate the angle in the FPGA is set to 3000, and the real-time calculation of the angle is output via the serial assistant after starting the acquisition. After the test, data with a time length of 83 s are generated. The test results and the real value of the angle are compared and analyzed. Figure 14 provides the comparison of the angle output results, where the blue trajectory shows the angle real-time output result after the designed method in this paper, the red trajectory shows real change range of the angle of the acoustic source relative to the sensor, which is −63 to −178°. The two angle change trajectories are close to each other. The indoor test results show that the detection system has the function of real-time angle output, and the angle estimation error is less than 3° when the noise is large.

### 3.2. Field Experiment

**Figure 15:**
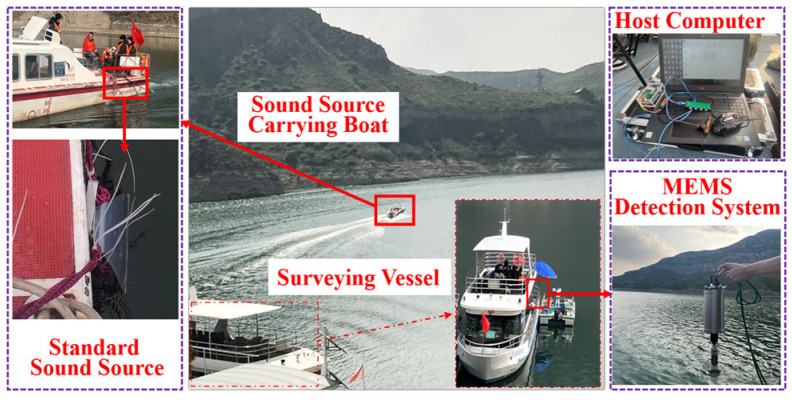
Reservoir experiment site.

The functions of the detection system are verified at a reservoir. Figure 15 shows the reservoir as the experimental site, the red annotations denote the names of the devices, with arrows indicating the magnified images of the equipment, while the purple boxes highlight the zoomed-in sections. The moving trajectory of the acoustic source vessel in the reservoir is detected by an underwater target orientation estimation MEMS hydroacoustic detection system. The system is suspended from the perimeter of the survey vessel, and the acoustic source vessel carries a fish-lip sounding transducer as a moving target. The experimental process is as follows: The target orientation estimation detection system is activated by the upper computer, and descends at a depth of 5 m underwater. When the acoustic source vessel arrives at a position about 70 m away from the receiving equipment, it gradually moves away from the detection system until the relative distance is 375 m, and transmits a 315 Hz sinusoidal signal while moving. The position of the survey vessel and the movement trajectory of the sound source vessel are recorded separately by a GPS logger, and the precise distance between the acoustic source vessel and the detection system is obtained by converting the position information of the two GPS locators.

**Figure 16:**
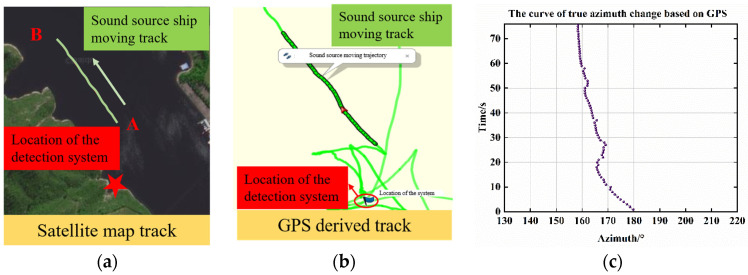
Trajectory of acoustic source ship movement: (**a**) GPS-derived trajectory; (**b**) satellite map trajectory; (**c**) angle change curve.

Figure 16a shows the satellite map of the experimental site, where A representing the initial position of the trajectory, B representing the final position of the trajectory, and star symbol indicating the positions of the test system. The target ship travels from point A to point B, and at the same time, the trajectory of the acoustic source movement is derived by the GPS locator installed on the target ship (as in Figure 16b) and the angular change curve of the target ship relative to the detection system is calculated and shown in Figure 16c. The GPS recording results show that the acoustic source vessel moves from the position 26 m away from the detection system to the position 86 m away, the average speed of the acoustic source ship is about 3 km/h, and the faster speed in the first 20 s is about 4 km/h. The angle changes within the range of 180.7–159.4°, and the angle span is 21.3°.

**Figure 17:**
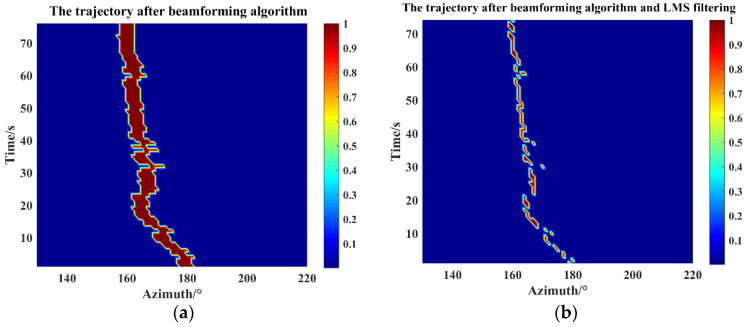
Calculation of acoustic source angle change based on raw data stored in the system: (**a**) after beamforming algorithm; (**b**) after beamforming algorithm and LMS filtering.

**Figure 18:**
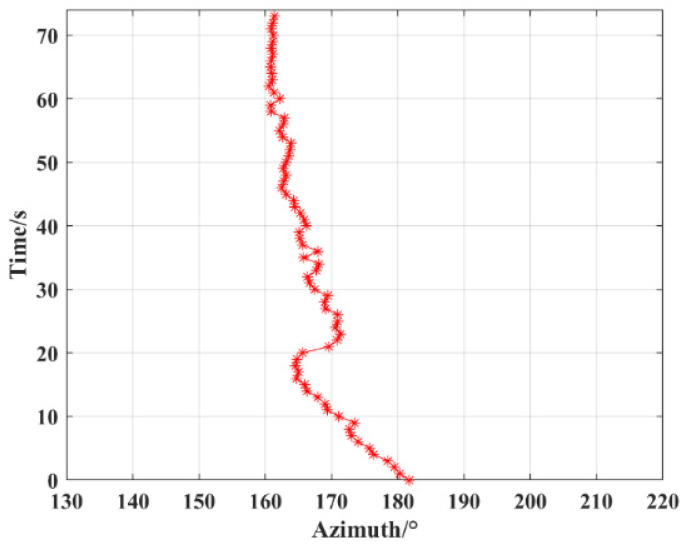
Simulation of real-time output results of DOA in reservoir experiments.

The target orientation information collected by the detection system is analyzed and processed to generate a data segment with a length of 74 s during the movement of the target ship. Firstly, the angle calculation results are analyzed by MATLAB, and the beamforming algorithm is used to calculate the moving trajectory of the acoustic source. Figure 17a shows the trajectory calculation results. It can be seen that the angle estimation error is large. The raw data are first calculated by the adaptive LMS filtering algorithm designed in this paper which then calculates the acoustic source moving trajectory by using the beamforming algorithm. Figure 17b shows the angle calculation results after filtering. It is clear that the angle estimation error is reduced, and there is a tremendous reduction in RMSE from 6.3890 to 2.4387 when compared to the angle obtained from the conversion of the GPS locator. In addition, the raw data recorded according to the system were input into Vivado 2018.3 for the optimized LMS adaptive filtering method as well as the simulation of the target orientation estimation algorithm, and the angular change is obtained as shown in Figure 18. The change interval is 181.6–161.4°, the angular change range is 20.2°, and RMSE is 2.6418 when compared to the actual angle. The results show that the MEMS underwater target orientation detection system can stably record the original signals for a long time and the recording results are reliable. In addition, the simulation of real-time hardware processing of raw data shows that the system is capable of real-time angle processing, and the output results are accurate and reliable.

Currently, Zhu Shan et al. have devised and executed a MEMS-based vector hydrophone system for underwater acoustic signal acquisition and storage, achieving a commendable standard [30]. They demonstrate the capability of precisely determining target orientations based on gathered signals. Through experimentation on DOA estimation at five distinct fixed locations, they have validated the DOA estimation proficiency of the self-contained acquisition system’s data collection and the amalgamated beamforming algorithm utilized. The maximum estimation discrepancy across different positions is delineated at 4.23°. Our equipment, compared to that of Zhu Shan et al., exhibits a maximum target estimation error of 3.1°, representing a reduction in estimation error of 1.13°. Notably, by embedding angle estimation and the improved LMS filtering method into the hardware system, obviating the need for post-processing raw data through a host computer, we not only enhance the directional precision but also eliminate the necessity for traditional host computer calculations, thus streamlining the data processing workflow. Additionally, the method of real-time outputting target angles instead of raw data significantly simplifies the demand for real-time data processing in subsequent applications, providing a basis for the precise real-time monitoring of the testing system. This innovation not only saves time and resources but also enhances system responsiveness, enabling users to swiftly acquire required information and make corresponding decisions.

## 4. Conclusions

A detection system for real-time estimation of underwater target orientation is presented in this paper. The MEMS vector sensors are applied to miniaturized marine target detection system for the first time, which greatly reduces the cost, power consumption, and volume of the detection system. The detection system can reliably store the original signal of the MEMS vector hydrophone in the internal SD card, such that the system is equipped with the monitoring and detection functions. Moreover, the adaptive signal processing and DOA estimation algorithm are integrated into the system, the simulation and experimental results in both indoor and reservoir show that this method can realize the real-time estimation of target orientation, and the estimation error is less than 3°. In this way, the method in this paper and the test results are of great significance for the estimation of marine target orientation. In conclusion, the MEMS marine target detection system proposed in this paper has unique features of low cost, small size, and high detection accuracy, becoming a miniaturized underwater target orientation estimation system representing good value for money. It has broad application prospects in the field of marine detection. Although the detection system is equipped with the functions of storage and real-time calculation, it is still necessary to transmit the real-time processing results to the upper computer via data lines. Therefore, integrating a wireless module into the system to communicate with the upper computer will be the focus of our future work.

## Figures and Tables

**Table 1:** Comparison of hydrophone performance.

Designer	Concavity Depth (dB)	Vector Channel Sensitivity (dB)	Gain Magnitude (dB)
Zhu et al. (2021 [2])	30	−182.7 dB@1 kHz (0 dB~1 V/µPa)	49.5
Geng et al. (2023 [18])	40	−180.9 dB@1 kHz (0 dB~1 V/µPa)	49.5
This Paper	40	−175.4 dB@1 kHz (0 dB~1 V/µPa)	54.0

**Table 2:** DOA estimation AVG, Sigma, and CI under different SNR.

SNR/dB	AVG/°	CI/°	Sigma	CI
−10	59.2259	[57.2809 61.1611]	9.7572	[8.5630 11.3295]
−5	60.0736	[59.5117 60.6351]	2.8307	[2.4854 3.7884]
0	59.9980	[59.8002 60.1958]	0.9968	[0.8752 1.1580]
5	60.0667	[59.9689 60.1644]	0.4925	[0.4324 0.5721]
10	59.9851	[59.9356 60.0346]	0.2494	[0.2190 0.2897]

## Data Availability

Data are contained within the article.
